# Alternative Living Kidney Donation Programs Boost Genetically Unrelated Donation

**DOI:** 10.1155/2015/748102

**Published:** 2015-09-02

**Authors:** Rosalie A. Poldervaart, Mirjam Laging, Tessa Royaards, Judith A. Kal-van Gestel, Madelon van Agteren, Marry de Klerk, Willij Zuidema, Michiel G. H. Betjes, Joke I. Roodnat

**Affiliations:** Department of Internal Medicine, Erasmus MC, s-Gravendijkwal 230, 3015 CE Rotterdam, Netherlands

## Abstract

Donor-recipient ABO and/or HLA incompatibility used to lead to donor decline. Development of alternative transplantation programs enabled transplantation of incompatible couples. How did that influence couple characteristics? Between 2000 and 2014, 1232 living donor transplantations have been performed. In conventional and ABO-incompatible transplantation the willing donor becomes an actual donor for the intended recipient. In kidney-exchange and domino-donation the donor donates indirectly to the intended recipient. The relationship between the donor and intended recipient was studied. There were 935 conventional and 297 alternative program transplantations. There were 66 ABO-incompatible, 68 domino-paired, 62 kidney-exchange, and 104 altruistic donor transplantations. Waiting list recipients (*n* = 101) were excluded as they did not bring a living donor. 1131 couples remained of whom 196 participated in alternative programs.* Genetically unrelated* donors (486) were primarily partners.* Genetically related* donors (645) were siblings, parents, children, and others. Compared to genetically related couples, almost three times as many genetically unrelated couples were incompatible and participated in alternative programs (*P* < 0.001). 62% of couples were genetically related in the conventional donation program versus 32% in alternative programs (*P* < 0.001). Patient and graft survival were not significantly different between recipient programs. Alternative donation programs increase the number of transplantations by enabling genetically unrelated donors to donate.

## 1. Introduction

More than 1 out of 4 donor-recipient couples are incompatible, because of either blood type ABO incompatibility or a positive crossmatch. These couples cannot participate in the conventional living donation program. Worldwide, alternative donation programs were developed to enable these couples to pursue donation and transplantation.

In kidney-exchange donation, a match is sought between 2 or more incompatible couples, so that each donor can donate to another couple's recipient [1–8]. In 10 years 632 couples were included in the Dutch national exchange program, with a 52% success rate [9].

For specified and unspecified altruistic donors there are several options. They can donate their kidney to a patient on the waiting list for deceased donor kidney transplantation, but they can also participate in the domino-paired donation program [10, 11]. In that program, the altruistic donor donates to the recipient of a couple that is unsuccessful in the national donor exchange program. The donor of that couple donates to the recipient of another incompatible couple or to a patient on the waiting list.

The ABO-incompatible donation program is the most beneficial program for blood type O recipients [8, 12–17]. In this program primarily blood type A donors donate to O recipients. The aim of these programs is to increase the number of transplantations carried out [18, 19]. Our study is on the relationship of the intended recipient with the willing donor that enabled participation in living donation programs, independent of eventual direct or indirect donation. This means that although there is a genetic relationship between a willing donor and an intended recipient, both may finally participate in genetically unrelated transplantations. We wondered what the influence of these alternative programs was on the composition of the donor pool: Do these programs actually increase the donor pool by enabling a new potential donor population to donate?

## 2. Methods

Between January 1, 2000, and January 1, 2014, 1935 transplantations have been carried out in Rotterdam: 703 deceased donor and 1232 living donor (LD) transplantations. In this retrospective cohort study, all first and repeat kidney transplant recipients of both conventional and alternative program LD kidney transplantations were included. In conventional transplantation and ABO-incompatible transplantation the willing donor donates directly to the intended, specified recipient. In kidney-exchange and domino-donation the willing donor donates indirectly to the intended recipient. All recipients who received a LD kidney while on the waiting list were excluded from this study as they did not bring a willing living donor. The character of the relationship between the intended, specified recipient and the willing LD that enabled participation in living donation programs was studied. Relationships were divided into genetically related and genetically unrelated ones. Genetically related donors were parents, siblings, children, and other family members who were second-degree blood relatives. Genetically unrelated donors were partners and other nonblood relatives. The latter group consisted of family by marriage and friends. In addition, reasons for participation in alternative donation programs were studied. These reasons could be blood type ABO incompatibility, positive crossmatch, or both or on a voluntary basis.

### 2.1. Statistical Analyses

We analysed the prevalence of different relationships between recipient-donor couples participating in conventional and alternative programs using chi-square tests. Univariate and multivariate Cox proportional hazards analyses were performed. Observation was until graft failure, until death, or until August 2015. The influence of program was tested as a variable with all programs separately and as a binary variable with conventional program versus all alternative programs pooled together. For multivariate analysis donor and recipient age and gender, pretransplant renal replacement treatment (yes or no), and preceding transplants (yes or no) were included. All analyses were performed using the Statistical Package for the Social Sciences (SPSS) 21.0.0.1 (IBM Corporation, Armonk, NY, USA). *P* values ≤0.05 were considered significant.

## 3. Results

**Figure 1:**
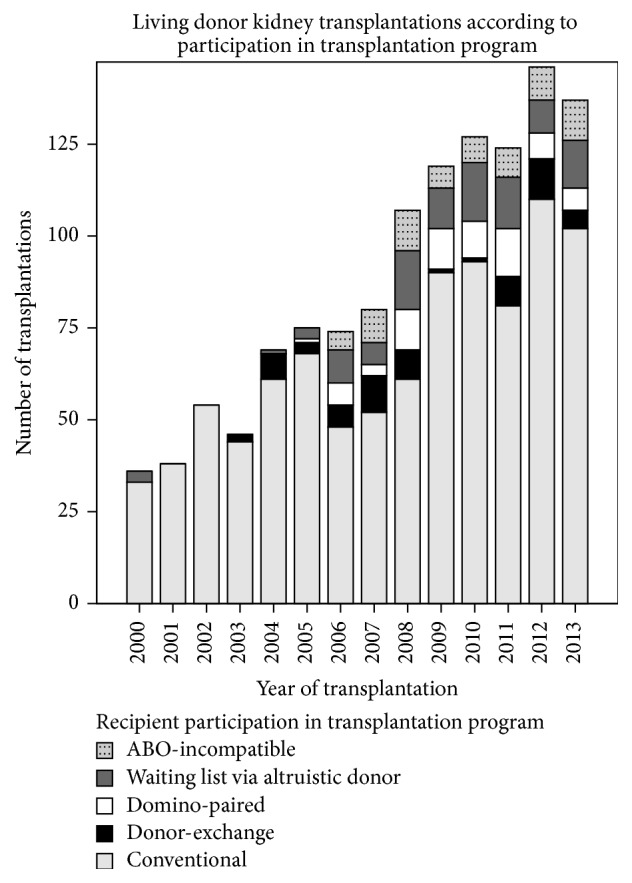
Number of living donor kidney transplantations in Rotterdam according to participation in transplantation program.

In Figure 1, the numbers of LD kidney transplantations carried out are shown. The light grey bars show numbers of conventional living donations; the other shades and fills represent alternative donation programs. A quarter of LD transplantations took place via an alternative program.

### 3.1. Participation in Alternative Program

Table 1 shows the participation of donors and recipients in the different programs. Horizontally, the recipients are shown and vertically the donors. Out of 1232 LD transplantations 935 were conventional and 297 were alternative program transplantations. Altruistic donors (*n* = 104) participated in the domino-paired program (*n* = 58) or donated to a recipient on the waiting list (*n* = 46). Domino donors (*n* = 65) donated to another domino recipient (*n* = 10) or to the waiting list (*n* = 55). There were 104 altruistic donors and only 101 waiting list recipients because 3 domino donors donated in other university hospitals in Netherlands.

### 3.2. Reasons for Alternative Program

There were 297 recipients of LD kidney transplantation via alternative donation programs; 101 of them were waiting list patients that did not bring a donor. The reasons for participation in alternative donation programs of the remaining 196 recipients were as follows: ABO incompatibility in 149 couples (76%), a positive crossmatch in 41 couples (21%), both ABO incompatibility and a positive crossmatch in 2 couples, and voluntary participation in 4 couples. Sixty-three incompatible couples were genetically related and 133 were genetically unrelated. In the population of genetically related incompatible couples 73% was ABO-incompatible and 22% had a positive crossmatch, while, in the genetically unrelated incompatible couple population, 77% was ABO-incompatible and 21% had a positive crossmatch (ns). All couples with both a positive crossmatch and ABO incompatibility were genetically related. Three out of four couples that participated voluntarily were genetically unrelated.

### 3.3. Donor-Recipient Relationship

The relationship between the intended, specified recipient and the willing LD that enabled participation in LD transplantation programs was studied. In the total population of donor-recipient combinations, including altruistic donor combinations (*n* = 1232), 48% of recipient and donor combinations were genetically unrelated and 52% were genetically related.

**Figure 2:**
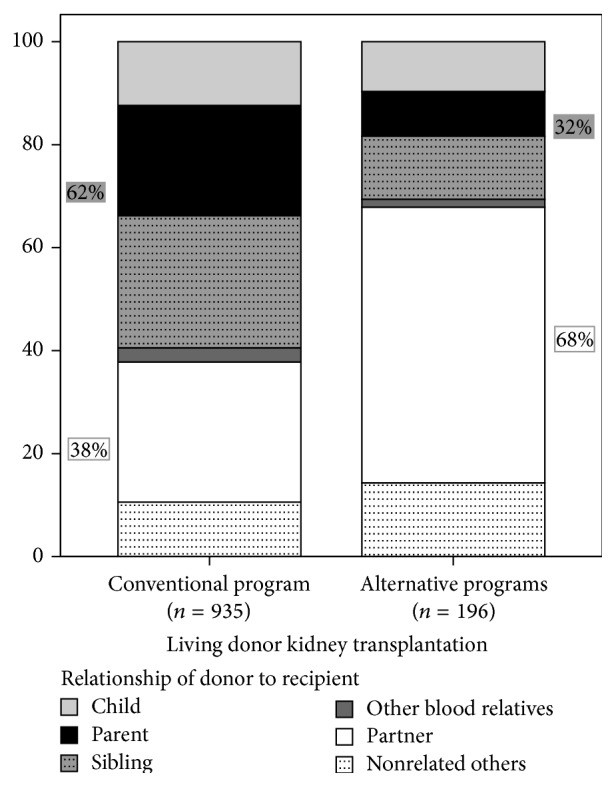
Distribution of donor-recipient relationships in conventional versus alternative living donor kidney transplantation programs. White fills = living unrelated and dark fills = living related couples (*P* < 0.001).

In the study on donor-recipient relationship, waiting list recipients were excluded, because they did not bring in a donor themselves (*n* = 101). 1131 couples remained of whom 196 couples participated in alternative living donation programs. There were 66 ABO-incompatible transplantations, 68 via domino-paired donation, and 62 via the local or national kidney-exchange programs (Table 1). In 1131 donor-recipient couples 43% were genetically unrelated and 57% genetically related.* Genetically unrelated* donors (*n* = 486) were partners (*n* = 359) or other nonblood relatives (*n* = 127).* Genetically related* donors (*n* = 645) were siblings (*n* = 264), parents (*n* = 217), children (*n* = 135), and other family members (*n* = 29).  Figure 2 shows donor-recipient relationships in conventional and alternative donation programs. In the conventional donation program 62% of couples were genetically related and 38% of couples were genetically unrelated. However, in alternative donation programs 32% of couples were genetically related and 68% of couples were genetically unrelated (*P* < 0.001). Conversely, only 10% of genetically related couples participated in alternative donation programs and 90% in the conventional living donation program, while 27% of nongenetically related couples participated in alternative donation programs and 73% in the conventional donation program (*P* < 0.001).

### 3.4. Survival Analyses

Observation was until August 10, 2015. In the period studied 176 graft failures and 127 deaths have been observed. The influence of the donor program with all programs separately in a categorical variable was tested. The program the patient participated in did not have a significant influence on patient death in univariate analysis. In multivariate analysis recipient age was the only variable with a significant influence on patient death. The program the patient participated in did not have a significant influence on graft failure in univariate analysis. In multivariate analysis of the influence on graft failure recipient age and donor age had a significant influence. Donor program did not have a significant influence on graft failure or patient death in multivariate analysis.

The binary variable containing conventional program (*n* = 935) versus all alternative programs together (*n* = 196) did not have a significant influence on graft failure or patient death in univariate or multivariate analysis either.

**Figure 3:**
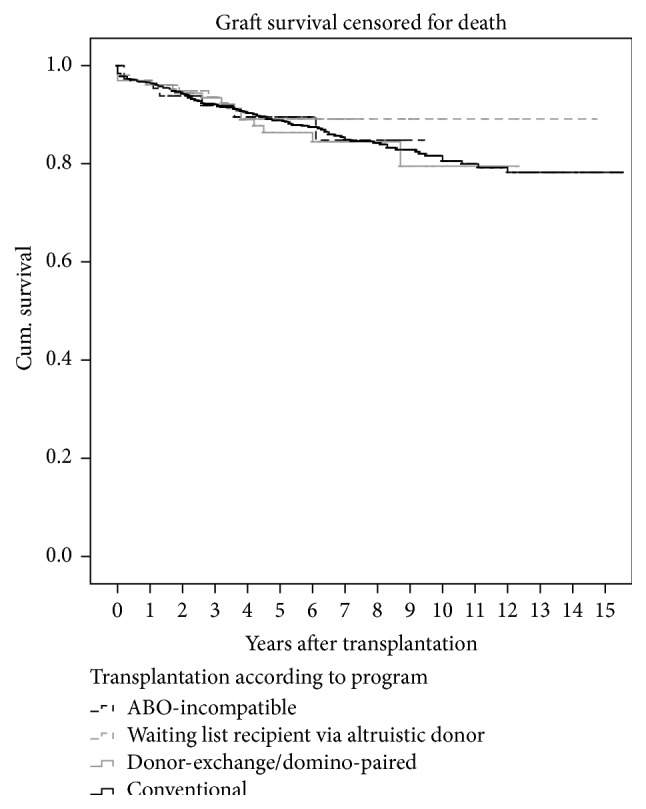
Graft survival censored for death for all living donor programs. Domino recipients and donor-exchange recipients are pooled.

Graft survival according to the programs the patients participated in is shown in the Kaplan-Meier curve (Figure 3). In order to increase numbers, the population of recipients via domino and donor-exchange programs are pooled as they are derived from the same population of incompatible couples.

## 4. Discussion

In 1995 the first report on living unrelated kidney donation showed that results were comparable to those of non-HLA identical living related transplants [20, 21]. Unrelated kidney donation is indispensable for those with hereditary kidney diseases like adult polycystic kidney disease. This may also hold true for recipients with some immunologic kidney disease as recent studies showed that genetically related donors who donated to recipients with immunologic disease run higher risks of developing kidney disease themselves [22, 23]. Since 1995 the numbers of unrelated living donor transplantations increased steadily in many nations [24]. According to Horvat the percentage of living unrelated donors varies between 10% (Mexico) and 75% (Saudi Arabia) of all living donors in the countries studied [24]. A high percentage of unrelated living donors are reported in Iran as this is the only country with a paid and regulated living unrelated kidney donation program [25]. Data of the Iranian national registry for kidney transplantation which comprises data of all renal transplantations performed in the country during a 22-year period were included in a study that revealed 14% living related and 86% living unrelated donors [26]. The percentage of unrelated donor transplantations is high in centers with flourishing donor-exchange, domino-donation, and/or ABO-incompatible transplantation programs compared to centers where conventional donation is the only option [4, 7, 10, 13, 22, 24, 27–32]. Our study is on the relationship of the intended recipient with the willing donor that enabled participation in living donation programs, independent of direct or indirect donation. In our population, in a period of 14 years, 48% of willing living donors were genetically unrelated to the recipient. After exclusion of combinations with altruistic donors, still 43% of couples were genetically unrelated. There is no paid and regulated living kidney donation program in Netherlands, but the spectrum of alternative living donation programs is a solid part of the transplantation program. Donation is on a voluntary and altruistic basis. The willingness of unrelated persons to donate a kidney differs between populations. Both in Europe and in the USA genetically unrelated donors are far more prevalent in Caucasian compared to African populations [33, 34]. Also, recipients living in poorer areas were more likely to receive a kidney from a genetically related donor and less likely from spouses or partners [31]. Apart from ethnicity and socioeconomic circumstances, donor-recipient incompatibility, which occurs more often in unrelated couples, may be an important cause of low numbers of unrelated donors. As we show in our study the prevalence of incompatibility is almost three times higher in unrelated couples but subdivision according to cause of incompatibility (ABO incompatibility or positive crossmatch) is not different compared to genetically related couples. This incompatibility as a reason for donor decline can be overcome by alternative donation programs. Our survival analyses and Figure 3 show that survival is not significantly different between conventional and alternative donation programs.

About 50% of incompatible couples that participate in the donor-exchange program have a positive crossmatch. However, about 20% of the population transplanted via an alternative transplantation program was incompatible with the intended donor because of a positive crossmatch. This means that incompatible couples with a positive crossmatch have a smaller chance to get a transplant via one of the current alternative transplantation programs compared to ABO-incompatible couples. As a matter of fact, of all living and deceased donor kidney transplantation programs in our center, most highly sensitized patients are transplanted via the deceased donor acceptable mismatch program [14, 35]. However, for some difficult-to-match phenotypes, desensitization serves as the only credible option [36, 37]. Expansion of the supply of alternative donation programs with a desensitization program is indispensable in order to increase the chances for this specific group of incompatible couples. Several experienced centers show good results of positive crossmatch transplantation using high dose IVIg and plasmapheresis [37–41]. This is the reason we recently started a transplantation program for couples with historically positive but currently negative crossmatches and a desensitization program was initiated recently.

In conclusion, alternative living donation programs increase the opportunity for nongenetically related couples to participate in living donation programs as they are more often incompatible with their intended recipient. Consequently, introduction and expansion of alternative living donation programs significantly expand the LD pool.

## Figures and Tables

**Table 1:** Participation of donor and recipient in the living donor transplantation programs.

Donor program	Recipient program					
	ABOi	Conventional	Domino	Waiting list	Donor-exchange	Total
ABOi	66	0	0	0	0	**66**
Altruistic	0	0	58	46	0	**104**
Conventional	0	935	0	0	0	**935**
Domino	0	0	10	55	0	**65**
Donor-exchange	0	0	0	0	62	**62**
Total	**66**	**935**	**68**	**101**	**62**	**1232**
